# The Supportive Effect of Acarbose to Orlistat in Weight Management—A Randomized, Double‐Blind, Multiarm Phase 2 Trial

**DOI:** 10.1002/oby.24369

**Published:** 2025-08-06

**Authors:** Ulf Holmbäck, Stefan Grudén, Sandra Kuusk, Helena Litorp, Joakim Englund, Arvid Söderhäll, Göran Alderborn, Anders Forslund

**Affiliations:** ^1^ Department of Public Health and Caring Sciences Uppsala University Uppsala Sweden; ^2^ Empros Pharma AB Solna Sweden; ^3^ Clinical Trial Consultants AB Uppsala Sweden; ^4^ Department of Women's and Children's Health Uppsala University Uppsala Sweden; ^5^ Department of Global Public Health Karolinska Institutet Stockholm Sweden; ^6^ Department of Pharmaceutical Biosciences Uppsala University Uppsala Sweden

## Abstract

**Objective:**

This study aimed to evaluate the added effect of acarbose to orlistat on relative weight loss in the novel antiobesity medication EMP16.

**Methods:**

In this 6‐month double‐blind trial, 240 individuals with obesity or overweight and comorbidities were randomized equally into three groups: (1) EMP16 (120 mg modified release orlistat/40 mg modified release acarbose), (2) MR‐O (120 mg modified release orlistat), and (3) Conv‐O (120 mg conventional orlistat). The primary outcomes were relative and categorical weight loss after 6 months.

**Results:**

Mean relative weight loss was −7.73% for the EMP16 group as compared to −5.78% for the MR‐O group and −5.13% for the Conv‐O group (*p* < 0.01). Proportion achieving ≥ 5% or ≥ 10% weight reduction was 61%/32% in EMP16 group, compared to 51%/20% in the MR‐O and 48%/12% in the Conv‐O group (*p* > 0.05 for ≥ 10%). All groups showed small improvements in glucose and lipid markers, with EMP16 demonstrating greater improvement in fatty liver index and quality of life compared to Conv‐O (*p* < 0.01). No serious adverse events occurred; most AEs were mild and transient.

**Conclusions:**

Acarbose enhances the weight loss efficacy of EMP16, supporting its potential as a safe and effective treatment for long‐term obesity management.

**Trial Registration:**

EU Clinical Trials Register: EudraCT‐nr. 2022‐003320‐40

**Study Importance**

- What is already known?
  ○ As obesity is a chronic disease, lifestyle modification often needs to be supplemented by weight loss medication.
  ○ Given that patients struggle to stay on current medications for a longer time, there is a need for a variety of treatment options.
- What does this study add?
  ○ In this 6‐month trial, adults with obesity were randomized to EMP16 (modified release combination of orlistat and acarbose), MR‐O (modified release orlistat), or Conv‐O (conventional orlistat).
  ○ The clinically relevant weight loss seen in a previous 6‐month study was confirmed with larger weight loss in the EMP16 group compared with the other groups.
  ○ Furthermore, the trial showed that the combination of orlistat and acarbose in EMP16 results in an effect on weight loss that is significantly larger than the effect of orlistat alone.
  ○ In addition to greater weight loss, EMP16 resulted in improvements in fatty liver index and quality of life.
- How might these results change the direction of research or the focus of clinical practice?
  ○ This trial shows that both orlistat and acarbose play a meaningful role in the novel combination antiobesity medication EMP16, and that the drug confers improvement in several health‐related domains.
  ○ EMP16 may be a suitable option for long‐term treatment of obesity.

## 1 Introduction

The advent of glucagon‐like peptide‐1 receptor agonists (GLP‐1RAs) and multiagonist therapies like semaglutide and tirzepatide has revolutionized obesity treatment, achieving unprecedented weight loss of 15%–25% [1]. However, about 40% of patients discontinue treatment within 3 months, rising to 50%–65% by 12 months in real‐world settings [1, 2]. The cost of treatment seems to be the major barrier for continued treatment, but lack of effect and to some degree gastrointestinal (GI) side effects (e.g., nausea, vomiting, constipation [3]) also are affecting attrition [2]. Therefore, there is a need to for a broad repertoire of antiobesity medications (AOMs) to help patients stay on treatment.

EMP16 is a modified release fixed‐dose combination of the lipase inhibitor orlistat and the alpha‐amylase inhibitor acarbose. Orlistat in its conventional dosage form (Xenical/Alli) has been used for decades and is still included in several obesity treatment guidelines [4], despite its modest effect of about 3% placebo‐corrected weight loss at 12 months [5]. Acarbose in its conventional dosage form (Precose), is indicated for the treatment of type 2 diabetes mellitus (T2DM) and shares orlistat's decades of long clinical use. However, acarbose in its conventional form is associated with minimal (≤ 1%) weight loss in participants with or without T2DM [6, 7].

EMP16 builds on the well‐established safety of orlistat and acarbose, and by modifying the release of orlistat and acarbose to distinct parts of the GI tract, the efficacy has been increased. In a previous phase 2 study, the achieved placebo‐corrected weight loss after 6 months of treatment was about 5%, with additional improvements in secondary outcome variables [8]. The weight loss achieved was larger than would have been expected using orlistat and acarbose in their conventional dosage forms in combination [9, 10]. Thus, the primary aim of this study was to confirm the added effect of acarbose in EMP16 on efficacy, that is, to show that acarbose has an additive and independent effect on relative weight loss. In addition, we wanted to compare EMP16‐120/40 to orlistat in its conventional dosage form, both for efficacy and safety.

## 2 Methods

### 2.1 Study Design

This was a randomized, double‐blind, active‐controlled phase 2 trial. The trial included a screening period of up to 5 weeks and a 26‐week treatment period. The trial had three main arms (see Section 2.3) and two smaller exploratory arms. These smaller arms will be presented in another publication. The trial was conducted by an independent clinical research organization (CRO), Clinical Trial Consultants AB, Uppsala, Sweden, in Linköping, Stockholm, and Uppsala. The protocol was approved by the local ethics committee in Stockholm, Sweden (Approval# Dnr. 2023‐00374‐01). Prior to any trial assessments, participants provided signed informed consent to participate in the trial. The trial was performed in accordance with the Declaration of Helsinki and ICH Good Clinical Practice, reported according to the Consolidated Standards of Reporting Trials (CONSORT) reporting guideline, and was registered in the EU Clinical Trials Register (EudraCT‐nr. 2022‐003320‐40).

### 2.2 Participants

Participants were recruited using the CRO database, advertisements in social media platforms, and radio. The trial population consisted of women and men aged between 18 and 75 years with a body mass index (BMI) of at least 30 kg/m^2^ or at least 27 kg/m^2^ in combination with other risk factors such as hypertension, glucose dysregulation (impaired glucose tolerance or T2DM), and/or dyslipidemia based on interview. Main exclusion criteria were T2DM treated with medication, a medical history that could affect the safety of the enrolled individual or the interpretation of trial results, and clinically significant findings in the physical examination, as well as clinically abnormal vital signs, electrocardiogram (ECG), or laboratory values at the time of screening as judged by the investigator. Full inclusion and exclusion criteria are listed in the online Supporting Information.

### 2.3 Procedures

The study procedures are described in detail in the online Supporting Information. In brief, the trial consisted of seven visits to the research units, including the screening visit. Participants arrived at the research clinic in the morning of the first dosing day (day 1, visit 2), and a reevaluation of eligibility including a brief physical examination, check of vital signs, and assessment of body weight was conducted before randomization. Blood sampling (fasting) and anthropometric measurements were performed. Lifestyle instructions were given as a short pamphlet where the key items were to eat a healthy diet with a maximum of 30% energy from fat and a low proportion of meal items with a high glycemic index (online Supporting Information). Participants returned to the clinic at week 4, 10, 18, and 26 for efficacy assessments of body weight and body composition and safety assessments including reporting of adverse events (AEs) and fasting blood sampling. In addition, phone calls were made in week 14, 18, and 22.

Participants were randomized to either of three main arms:

1. EMP16‐120/40 (modified release combination of 120 mg orlistat/40 mg acarbose);
2. MR‐O (modified release orlistat 120 mg);
3. Conv‐O (orlistat 120 mg in its conventional dosage form, Xenical).

Sex was used as a stratification variable to ensure a roughly equal male/female ratio in each treatment arm. The randomization list was generated by the CRO using SAS version 9.4 (SAS Institute Inc.), contained participant number and treatment, and was kept by the randomizer in a sealed envelope until database lock. Individual packaging and labeling of the IMP were performed by Créapharm Développement, le Haillan, France, based on the list.

### 2.4 Outcomes

Primary outcome variables were relative weight loss after 26 weeks of treatment and the proportion of participants losing at least 5% of their baseline body weight. A number of secondary outcome variables were also assessed (see online Supporting Information for more details): proportion of participants losing at least 10% of their baseline body weight, BMI, waist circumference, sagittal abdominal diameter, body fat percentage, blood pressure, heart rate, fasting total, LDL, and HDL cholesterol, ApoA1, ApoB, triglycerides, glucose, insulin, HbA1c, albumin, hs‐CRP, and the liver enzymes aspartate aminotransferase (AST), alanine aminotransferase (ALT), alkaline phosphatase (ALP), and gamma‐glutamyl transferase (GGT).

The homeostatic model assessment [HOMA] index, visceral adiposity index (VAI), and fatty liver index (FLI) were calculated. VAI is a composite index encompassing waist circumference, BMI, triglycerides, and HDL [11]. FLI is a composite index encompassing triglycerides, BMI, GGT, and waist circumference [12, 13].

Questionnaires used were: RAND‐36, EQ‐5D‐5L, GAD‐7, PHQ‐9, and TFEQ (online Supporting Information). Participants were also asked about diet, sleep, and physical activity during the trial.

### 2.5 Statistics

The number of participants was based on an assumed dropout rate of 15% (based on previous phase 2 trial [8]). A total of 80 participants had to be randomized to each of the three main treatment arms (EMP16‐120/40, MR‐O, and Conv‐O) to achieve at least 68 evaluable participants per arm, providing 80% power to detect a 2% difference in relative weight loss. Continuous data were analyzed using a mixed model for repeated measures (MMRM). The model included all trial treatments and all visits. Categorical data were analyzed pairwise for all comparisons using a chi‐square test for difference in sample proportions. Change from baseline in quality of life (RAND‐36 and EQ‐5D‐5L) was analyzed using multi‐way analysis of variance (ANOVA). No statistical testing was performed for GAD‐7, PHQ‐9, TFEQ, or for lifestyle questions. Adverse events of special interest, oily spotting and fecal incontinence, were tested with mixed effects logistic regression with repeated measures analysis. Alpha‐level 5% was used in all hypothesis testing. The principal inferential statistical analyses in this trial were performed using various applications of mixed models and the response data were assumed to be missing at random (MAR). An additional multiple imputation (MI) analysis was done for some models. All descriptive summaries and statistical analyses were performed using SAS version 9.4.

## 3 Results

**FIGURE 1:**
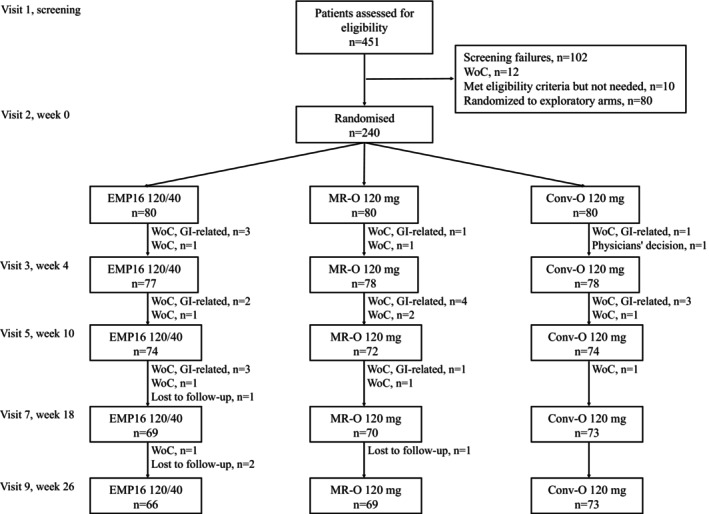
Participant flowchart. WoC, withdrawal of consent; EMP16, modified release combination of 120 mg orlistat/40 mg acarbose; MR‐O, modified release orlistat 120 mg; and Conv‐O, orlistat 120 mg in its conventional dosage form. Visit 4, 6 and 8 were phone check‐ups.

A total of 451 potential participants were screened, and 240 were randomized to the three main arms and 80 to the smaller exploratory arms. The smaller exploratory arms (half‐dose EMP16 and placebo) will be presented in a separate publication. Of the 208 who completed the trial (Figure 1), 107 were females and 101 males. Almost all participants (95%) described themselves as non‐Hispanic White and the ratio “Current+Former/Never” for nicotine use was 37/53, 32/48, and 51/39 for EMP16, MR‐O, and Conv‐O participants, respectively. In an explorative post hoc Fisher exact test, the proportion tended (*p* = 0.052) to be different between EMP16 and Conv‐O participants. Hypertension was reported by 16%, 28%, and 23% of EMP16, MR‐O, and Conv‐O participants, respectively, whereas other metabolic diseases were reported by less than 5% of the participants. Additional baseline characteristics are displayed in Table 1.

**TABLE 1:** Baseline characteristics, mean (SD) for continuous variables and median (Q1, Q3) for HOMA and categorical variables.

Assessment (unit)	EMP16 (*n* = 80)	MR‐O (*n* = 80)	Conv‐O (*n* = 80)
Age (years)	44.9 (11.1)	46.0 (10.6)	46.1 (10.7)
Sex (female/male)	41/39	38/42	40/40
Weight (kg)	104.0 (15.6)	106.7 (20.3)	105.7 (16.9)
BMI (kg/m^2^)	35.3 (4.0)	35.2 (4.8)	35.5 (4.1)
Waist circumference (cm)	112.7 (10.7)	114.6 (13.5)	115.5 (11.7)
Sagittal abdominal diameter (cm)	26.9 (2.9)	27.0 (3.6)	27.5 (3.0)
Body fat (%)	39.6 (7.1)	38.5 (7.0)	40.2 (6.8)
Systolic blood pressure (mmHg)	135.8 (12.2)	135.0 (12.7)	133.1 (13.1)
Diastolic blood pressure (mmHg)	85.2 (7.5)	84.8 (7.0)	84.5 (8.0)
Pulse (beats/min)	69.1 (10.8)	68.2 (10.3)	70.0 (11.5)
Fasting glucose (mg/dL)	99.46 (14.16)	98.67 (12.86)	100.34 (11.07)
Fasting insulin (mIU/L)	10.13 (4.84)	11.53 (7.26)	13.47 (13.66)
HbA1c (%)	5.34 (0.32)	5.36 (0.29)	5.36 (0.35)
HOMA	2.40 (1.60, 3.00)	2.40 (1.80, 3.50)	2.75 (2.00, 3.85)
Total cholesterol (mg/dL)	197.4 (40.9)	198.5 (36.5)	204.1 (37.1)
HDL (mg/dL)	48.6 (13.0)	50.4 (16.2)	47.5 (12.1)
LDL (mg/dL)	138.8 (38.4)	137.6 (33.5)	140.76 (29.9)
ApoB (mg/dL)	101.6 (25.1)	100.5 (22.7)	103.9 (19.3)
Triglycerides (mg/dL)	117.0 (57.3)	118.0 (60.9)	132.3 (61.5)
Visceral adiposity index	1.92 (1.09)	1.91 (1.24)	2.26 (1.34)
Fatty liver index	81.7 (17.7)	80.5 (17.9)	85.9 (15.0)
Physical functioning	85.0 (70.0, 95.0)	82.5 (70.0, 90.0)	85.0 (75.0, 90.0)
Role functioning/physical	100.0 (50.0, 100.0)	100.0 (75.0, 100.0)	100.0 (50.0, 100.0)
Pain (bodily pain)	80.0 (57.5, 90.0)	77.5 (67.5, 90.0)	80.0 (57.5, 90.0)
General health	70.0 (60.0, 80.0)	65.0 (50.0, 80.0)	70.0 (55.0, 80.0)
Energy/fatigue (vitality)	55.0 (40.0, 75.0)	55.0 (40.0, 70.0)	60.0 (42.5, 70.0)
Social functioning	87.5 (62.5, 100.0)	81.3 (62.5, 100.0)	87.5 (75.0, 100.0)
Role functioning/emotional	100.0 (66.7, 100.0)	100.0 (66.7, 100.0)	100.0 (66.7, 100.0)
Emotional well‐being (mental health)	76.0 (68.0, 88.0)	76.0 (68.0, 88.0)	76.0 (68.0, 88.0)
Health transition	50.0 (50.0, 62.5)	50.0 (50.0, 50.0)	50.0 (50.0, 50.0)
EQ‐5D‐5L, your health today (VAS)	70.5 (59.5, 80.0)	70.0 (51.0, 80.0)	70.0 (50.0, 78.0)
EQ‐5D‐5L, combined score	7.0 (6.0, 8.5)	7.0 (6.0, 9.0)	7.0 (6.0, 8.0)

**FIGURE 2:**
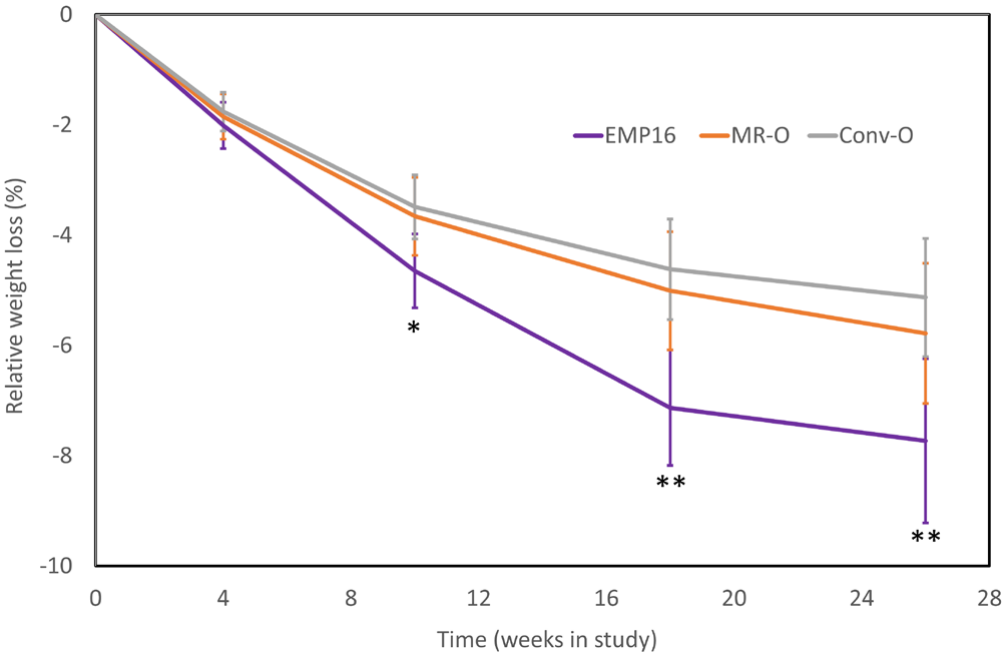
Change in body weight. Data displayed are mean percent weight loss from baseline (95% CI) without imputation for missing values. *p < 0.05 versus Conv‐O, **p < 0.01 versus MR‐O and Conv‐O, mixed model for repeated measures using multiple imputation for missing data. [Color figure can be viewed at wileyonlinelibrary.com]

Mean relative weight loss at week 26 was larger (7.73%) for EMP16 participants compared with MR‐O (5.78%, *p* = 0.004) and Conv‐O (5.13%, *p* = 0.002) participants (Figure 2). Sixty‐one percent of EMP16 participants lost ≥ 5% in body weight at week 26 compared to participants on MR‐O (51%, *p* = 0.059) and Conv‐O (48%, *p* = 0.086, Table 2). A higher proportion of participants on EMP16 (32%) had lost ≥ 10% in body weight at week 26 compared to participants on MR‐O (20%, *p* = 0.037) and Conv O (12%, *p* = 0.004) (Table 2).

**TABLE 2:** Primary and secondary outcome variables, changes from baseline to week 26.

	EMP16 (*n* = 66)	MR‐O (*n* = 69)	Conv‐O (*n* = 73)	Estimated difference (95% CI)^b^	*p*	Estimated difference (95% CI)^b^	*p*
	Mean (95% CI)^a^	Mean (95% CI)^a^	Mean (95% CI)^a^	EMP16 vs. MR‐O	EMP16 vs. MR‐O	EMP16 vs. Conv‐O	EMP16 vs. Conv‐o
Relative weight change (%)	−7.73 (−9.18 to −6.28)	−5.78 (−7.03 to −4.53)	−5.13 (−6.18 to −4.08)	−2.55 (−4.30 to −0.80)	0.004	−2.73 (−4.47 to −0.99)	0.002
Proportion of participants with ≥ 5% weight loss	61%	51%	48%	—	0.059	—	0.086
Proportion of participants with ≥ 10% weight loss	32%	20%	12%	—	0.037	—	0.004
Weight (kg)	−7.95 (−9.22 to −6.25)	−6.14 (−7.05 to −4.51)	−5.46 (−6.20 to −4.06)	−2.56 (−4.44 to −0.67)	0.008	−2.69 (−4.6049 to −0.77)	0.006
BMI (kg/m^2^)	−2.72 (−3.26 to −2.18)	−2.05 (−2.50 to −1.61)	−1.86 (−2.27 to −1.46)	−0.90 (−1.53 to −0.27)	0.005	−0.93 (−1.54 to −0.31)	0.004
Body fat (%)	−2.51 (−3.32 to −1.69)	−2.11 (−2.85 to −1.38)	−1.93 (−2.56 to −1.30)	−1.10 (−2.22 to 0.01)	0.053	−1.26 (−2.38 to −0.14)	0.027
WC (cm)	−8.10 (−9.91 to −6.28)	−6.01 (−7.50 to −4.52)	−5.51 (−6.80 to −4.21)	−2.28 (−4.41 to −0.15)	0.036	−2.44 (−4.57 to −0.32)	0.0244
SAD (cm)	−2.60 (−3.22 to −1.98)	−2.24 (−2.80 to −1.67)	−1.87 (−2.39 to −1.35)	−0.66 (−1.42 to 0.09)	0.087	−0.95 (−1.72 to −0.19)	0.015
Systolic BP (mmHg)	−3.5 (−6.5 to −0.4)	−1.4 (−3.8 to 1.0)	−1.7 (−4.3 to 0.9)	−1.96 (−5.22 to 1.30)	0.238	−0.98 (−4.34 to 2.38)	0.567
Diastolic BP (mmHg)	−1.3 (−3.3 to 0.6)	−1.5 (−3.1 to 0.2)	−0.3 (−2.1 to 1.5)	−0.06 (−2.34 to 2.22)	0.959	−1.11 (−3.35 to 1.13)	0.332
Pulse (beats/min)	−3.8 (−5.8 to −1.8)	−1.2 (−3.2 to 0.7)	−0.5 (−2.5 to 1.6)	−2.5 (−5.0 to −0.0)	0.047	−3.3 (−5.9 to −0.8)	0.009
Glucose (mg/dL)	−4.41 (−6.6 to −2.23)	−3.82 (−5.63 to −2.00)	−1.88 (−3.59 to −0.16)	−0.14 (−2.38 to 2.66)	0.914	−1.83 (−4.34 to 0.65)	0.152
HbA1c (%)	−0.14 (−0.19 to −0.10)	−0.15 (−0.20 to −0.10)	−0.16 (−0.21 to −0.11)	0.02 (−0.04 to 0.09)	0.525	0.03 (−0.04 to 0.09)	0.425
Insulin (mIU/L)	−2.47 (−3.56 to −1.38)	−2.31 (−3.36 to −1.26)	−2.68 (−3.77 to −1.60)	0.91 (0.79 to 1.04)^c^	0.163	0.89 (0.77 to 1.03)	0.109
HOMA	−0.72 (−1.03 to −0.41)	−0.67 (−0.99 to −0.35)	−0.71 (−1.00 to −0.41)	0.91 (0.78 to 1.07)^c^	0.261	0.88 (0.75 to 1.03)	0.101
Total cholesterol (mg/dL)	−17.99 (−25.26 to −10.72)	−17.97 (−23.74 to −12.20)	−17.00 (−23.23 to −10.78)	−0.40 (−8.73 to 7.94)	0.926	−2.40 (−10.64 to 5.85)	0.569
LDL (mg/dL)	−12.95 (−19.64 to −6.26)	−11.54 (−17.00 to −6.09)	−12.61 (−17.85 to −7.37)	−0.73 (−7.97 to 6.50)	0.843	1.18 (−5.60 to 8.36)	0.747
ApoB (mg/dL)	−7.32 (−11.86 to −2.78)	−8.35 (−11.51 to −5.20)	−6.77 (−9.92 to −3.61)	1.01 (−3.73 to 5.91)	0.657	−0.91 (−5.73 to 3.92)	0.713
HDL (mg/dL)	−4.80 (−6.47 to −3.12)	−2.56 (−4.02 to −1.11)	−3.40 (−5.16 to −1.63)	−2.26 (−4.27 to −0.25)	0.027	−0.74 (−2.79 to 1.31)	0.479
TG (mg/dL)	−9.66 (−21.23 to 1.91)	−13.43 (−24.09 to −2.77)	6.08 (−6.82 to 18.98)	1.01 (0.86 to 1.15)^c^	0.911	0.83 (0.73 to 0.94)	0.003
VAI	0.01 (−0.24 to 0.26)	−0.13 (−0.34 to 0.08)	0.16 (−0.06 to 0.39)	1.06 (0.95 to 1.23)^c^	0.444	0.84 (0.72 to 0.96)^c^	0.014
FLI	−15.15 (−19.91 to −10.38)	−10.71 (−14.24, −7.187)	−6.12 (−8.541, −3.704)	−5 (−10 to −0.1)	0.047	−9 (−14 to −4)	< 0.001
Physical functioning	9.6 (6.3 to 12.9)	9.6 (6.5 to 12.7)	4.6 (0.9 to 8.3)	−0.76 (−5.00 to 3.44)	0.722	4.54 (0.4003 to 8.67)	0.032
Role functioning/physical	13.3 (4.6 to 21.9)	6.9 (−0.2 to 13.9)	7.2 (−2.5 to 16.8)	0.86 (−8.50 to 10.22)	0.857	4.71 (−4.62 to 14.02)	0.323
Pain (bodily pain)	6.6 (0.7 to 12.4)	6.3 (1.7 to 10.8)	4.0 (−0.6 to 8.6)	−0.624 (−6.91 to 5.66)	0.846	2.02 (−4.03 to 8.08)	0.513
General health	5.3 (1.1 to 9.5)	9.3 (5.3 to 13.2)	1.6 (−1.7 to 4.9)	−3.17 (−7.85 to 1.59)	0.194	2.72 (−1.75 to 7.19)	0.233
Energy/fatigue (vitality)	4.8 (0.6 to 9.0)	8.6 (3.8 to 13.3)	1.1 (−3.1 to 5.3)	−2.79 (−8.31 to 2.74)	0.323	2.77 (−2.73 to 8.27)	0.323
Social functioning	3.8 (−1.7 to 9.3)	8.3 (3.3 to 13.4)	0.7 (−4.5 to 5.9)	−3.25 (−9.35 to 2.84)	0.295	0.23 (−5.75 to 6.22)	0.939
Role functioning/emotional	2.0 (−5.7 to 9.7)	9.2 (0.5 to 17.9)	4.1 (−5.7 to 13.9)	−3.19 (−13.64 to 7.27)	0.550	−0.74 (−10.84 to 9.34)	0.886
Emotional well‐being (mental health)	1.7 (−1.7 to 5.1)	2.8 (−0.7 to 6.3)	0.8 (−2.4 to 3.9)	−1.14 (−5.45 to 3.17)	0.603	0.37 (−3.75 to 4.48)	0.861
Health transition	23.1 (16.8 to 29.4)	19.6 (12.7 to 26.5)	14.0 (8.3 to 19.8)	7.8 (0.9 to 14.7)	0.026	9.2 (2.2 to 16.1)	0.010
EQ‐5D‐5L, your health today (VAS)	6.6 (2.8 to 10.5)	7.4 (3.3 to 11.6)	4.2 (0.4 to 8.1)				
EQ‐5D‐5L combined Score	−0.6 (−1.1 to −0.1)	−0.6 (−1.0 to −0.2)	−0.3 (−1.0 to 0.3)	0.1 (−0.5 to 0.7)	0.768	−0.9 (−0.2 to 0.4)	0.460

Abbreviations: FLI: fatty liver index; HbA1c: glycosylated hemoglobin A1C; HDL: high density lipoprotein; LDL low density lipoprotein; SAD: sagittal abdominal diameter; VAI: visceral adiposity Index.

^a^ Observed values without imputation.

^b^ Pairwise treatment comparisons based on mixed model for repeated measures (MMRM), using multiple imputation of missing data.

^c^ Modeled on natural log scale to improve model fit diagnostics. The results have been back‐transformed to present geometric means ratios of test versus reference treatment.

Participants on EMP16 had larger (*p* < 0.05 for all variables) absolute reductions from baseline in weight, BMI, waist circumference, sagittal abdominal diameter, and percentage of body fat at week 26 compared to Conv‐O participants (Table 2). Additionally, participants on EMP16 had larger (*p* < 0.05) absolute reductions in weight, BMI, and waist circumference at week 26 compared to MR‐O participants (Table 2).

Fasting glucose and lipid metabolism markers generally decreased in all treatment groups between baseline and week 26. For most comparisons, there were no clinically relevant differences between treatment groups in the change from baseline to week 26 in glucose metabolism and lipid metabolism markers (Table 2). Participants on EMP16 had a lower VAI compared to participants on Conv‐O at the end of the trial (Table 2, *p* = 0.014).

Participants on EMP16‐120/40 had a larger decrease in FLI compared to participants on MR‐O (*p* = 0.047) and Conv‐O (*p* < 0.001) (Table 2).

There were no differences between treatment groups in terms of change from baseline to week 26 in mean systolic and diastolic blood pressure (Table 2). Participants on EMP16 had larger reductions in pulse rate compared to participants on MR‐O (*p* = 0.047) and Conv‐O (*p* = 0.009) between baseline and week 26 (Table 2).

Larger improvements were observed in EMP16 participants compared with Conv‐O participants in the RAND‐36 domains “physical function” (*p* = 0.032) and “health transition score” (*p* = 0.010) (Table 2). In addition, improvements were observed in 4 of the 7 other RAND‐36 domains in the EMP16 group (Table 2). Improvements in several domains were also observed in the MR‐O group, but not in the Conv‐O group (Table 2). Health‐related improvements in quality of life based on the EQ‐5D‐5L instrument were in line with those obtained based on RAND‐36 (Table 2).

Self‐reported meal patterns improved during the study in all groups (Table S2), with the largest differences being decreasing intake of sweets and fast food, increasing intake of fiber‐rich products and eating three meals a day (Table S3). No change in amount or intensity of physical activity or changes in sleep characteristics were reported (Table S2).

Participants seemed to have normal eating behavior based on the TFEQ, and only minor changes were seen during the trial (Table S4).

Most participants in the trial did not suffer from anxiety or depression based on the GAD‐7 and PHQ‐9 questionnaires and there were no apparent differences between treatment groups in terms of change from baseline to week 26 in these aspects (Table S5).

No SAEs occurred and the AEs were mostly (88%) reported as mild, with the majority (65%) being GI events (Table 3, Table S6). There were no significant differences between the treatment groups either in terms of overall dropout rate or GI‐related withdrawals (Table 3). Overall prevalence of oily spotting was similar in EMP16 participants compared to MR‐O (*p* = 0.092) and Conv‐O (*p* = 0.428) participants (Table S7). Overall prevalence of fecal incontinence was similar in EMP16 participants compared to MR‐O (*p* = 0.108), and Conv‐O participants (*p* = 0.066) (Table S7). Both AEs of special interest had an increased prevalence in the beginning of the trial (Table S7). All mean ALT, AST, ALP, and GGT values in the treatment groups were within normal reference ranges throughout the trial (Table S8).

**TABLE 3:** Withdrawal and adverse events (AEs) with a prevalence of at least 5% in any group.

	EMP16		MR‐O		Conv‐O	
	*n* (%)	*m*	*n* (%)	*m*	*n* (%)	*m*
Overall withdrawal	14 (18%)		11 (14%)		7 (8.8%)	
Withdrawal due to GI‐AEs	7 (8.8%)		6 (7.5%)		4 (5.0%)	6
Total AEs	78 (98%)	334	73 (91%)	277	75 (94%)	281
Mild severity^a^	76 (95%)	288	71 (89%)	248	74 (93%)	246
Moderate severity	29 (36%)	46	19 (24%)	27	26 (33%)	34
Severe severity	0	0	2 (2.5%)	2	1 (1.3%)	1
Diarrhea	50 (63%)	57	51 (64%)	67	53 (66%)	58
Flatulence	46 (58%)	49	24 (30%)	24	15 (19%)	15
Nasopharyngitis	23 (29%)	25	32 (40%)	40	26 (33%)	31
Oily spotting^b^	33 (41%)	38	22 (28%)	26	25 (31%)	29
Fecal incontinence^b^	19 (24%)	22	10 (13%)	12	11 (14%)	11
Steatorrhea	15 (19%)	15	4 (5.0%)	4	10 (13%)	10
Abdominal distension	13 (16%)	15	9 (11%)	9	5 (6.3%)	7
Headache	13 (16%)	13	7 (8.8%)	8	8 (10%)	11
Defecation urgency	3 (3.8%)	3	4 (5.0%)	5	8 (10%)	11
Abdominal pain	5 (6.3%)	7	6 (7.5%)	6	5 (6.3%)	5
Abdominal pain upper	4 (5.0%)	7	5 (6.3%)	5	6 (7.5%)	6
Nausea	4 (5.0%)	4	1 (1.3%)	1	5 (6.3%)	5
Back pain	2 (2.5%)	2	4 (5.0%)	4	1 (1.3%)	1
COVID‐19	0	0	4 (5.0%)	4	1 (1.3%)	1
Decreased appetite	1 (1.3%)	1	4 (5.0%)	4	1 (1.3%)	1

Abbreviations: *m*, number of events; *n*, number of participants.

^a^ The grading of the severity/intensity (grade 1 to grade 5) of AEs followed the common terminology criteria for AEs. No grade 4 or 5 events occurred.

^b^ Oily spotting is equivalent to the preferred term “rectal discharge” and fecal incontinence is equivalent to the preferred term “anal incontinence.”

## 4 Discussion

In this trial, treatment with EMP16 for 26 weeks led to a larger weight loss compared with MR‐O and Conv‐O. Other anthropometric measurements such as BMI, waist circumference, sagittal abdominal diameter, and percentage of body fat confirmed similar treatment effects of EMP16 seen in a previous trial [8]. Small improvements in glucose metabolism markers and blood lipids were observed in all treatment groups. Patient‐reported quality of life improvements were larger in the EMP16 group compared with Conv‐O. All participants reported improvements in meal pattern, but there was no other indication of lifestyle change. No SAEs occurred and the majority of AEs were mild.

The relative weight loss in the EMP16 group was 7.7%, whereas the MR‐O group had a weight loss of 5.8% and the Conv‐O group 5.1%. This shows that acarbose has a meaningful additive and independent effect on weight loss, and that EMP16 was superior to Conv‐O in terms of weight loss. The effects on weight and other anthropometric parameters were in line with those obtained for EMP16 in a previous trial [8]. Acarbose has previously been shown to have a small (≤ 1%) weight loss effect, regardless of the presence of diabetes [6, 7, 9]. In this trial, the acarbose component was responsible for 25% of the weight loss effect, despite the low dose of acarbose employed. Possibly, the modified release pattern in EMP16 substantially enhanced acarbose efficiency, in line with the study by O'Dea et al. [14], where mixing of acarbose with food improved postprandial effects compared to its conventional dosage form.

The design of the SESAM study and its impact on weight loss need a brief comment. The SESAM study, as well as the previous study [8], had a very limited lifestyle component, without any caloric goals or dietitian support and a limited number of visits. The impact of the lifestyle component and visit frequency can be exemplified by comparing two trials using semaglutide 2.4 mg as target dose. In the STEP‐1 trial, the participants were advised to eat a reduced caloric diet, and participants had frequent meetings providing support. In the SELECT trial, the participants were instructed to eat a healthy diet without caloric restriction, and the number of visits was much lower [15]. At 26 weeks, the average weight loss was about 12% in the STEP‐1 trial [16] and about 7% in the SELECT trial [15]. Possibly, a more intensive lifestyle intervention in the current trial could have had an impact on achieved weight loss. The participants did report a decreased intake of sweets and fast food and increased intake of fiber‐rich products, possibly indicating a shift toward healthier diets; but we have no data on the magnitude of these changes.

Even though there were no statistical differences in anthropometric variables between MR‐O and Conv‐O, a small but over time possibly clinically relevant effect of the modified release pattern cannot be ruled out as MR‐O consistently showed 10%–20% larger improvements in several outcome variables.

There were no major differences in glucose or lipid metabolism markers between the treatment groups despite differences in weight loss. This is line with other studies where participants do not show signs of glucose dysregulation at baseline. In the STEP 1 trial mentioned earlier [16], the ETD in HbA1c was only 0.29%, and in the SURMOUNT‐1 trial [17] the difference between the 15 mg tirzepatide group and placebo was only 0.44%, despite the large differences in weight loss seen in these trials. Still, the small signs of improvement in glucose metabolism are in line with the decreased T2DM incidence observed with conventional orlistat and acarbose [7, 18]. The mean LDL concentration decreased by ≈10% (≈12mg/dL), and about 40% of the participants had a ≥ 20mg/dL decrease in LDL between baseline and week 26. That orlistat has a clinically meaningful effect on LDL concentration has been shown before [10], and its impact on blood lipids is somewhat larger than other AOMs [16, 19, 20].

The estimated prevalence of metabolic dysfunction‐associated steatotic liver disease (MASLD) decreased more in the EMP16 group compared to the Conv‐O group, as judged by the FLI [12]. The FLI has been shown to have a good association with more direct measures of liver composition [13]. At baseline, about 3% of all enrolled participants had a FLI below the suggested presence of MASLD cutoff (30 for women and 60 for men) [13]. At week 26, more than 20% of the EMP16 participants had a FLI below the cutoff for MASLD but only 4% of the participants in the Conv‐O group. Even though weight loss by itself has been associated with decreased hepatic fat content, the difference in FLI was larger than a pure weight loss effect as FLI dropped with 18% in the EMP16 group and 7% in the Conv‐O group. In a previous trial [21], the glucagon/insulin ratio [22] was higher in the EMP16 group compared with Conv‐O, suggesting a possible mechanism by which EMP16 may have a larger positive effect on liver fat.

In line with the previous trial [8], participants in the EMP16 group reported improved quality of life. Clinically relevant improvements [23] were observed in five of the eight domains in the EMP16 group and the health transition score increased by almost 40%. This is in contrast to the Conv‐O group where few improvements were seen and the increase in health transition was more modest. Interestingly, the improvements in quality of life seem to be larger than what have been reported for semaglutide and tirzepatide [16, 17], even when considering that those studies used SF‐36 and RAND‐36 has been used in this and the previous study. The reasons for the large improvement in quality of life seen in both studies are not entirely clear.

The vast majority of the AEs were assessed as mild in intensity. GI‐related events were the most common types of AEs in all treatment groups and were reported by 85% to 90% of the participants. Even though overall prevalence was not statistically significant different, GI‐AEs were more prevalent in the EMP16 group in the initial part of the trial. This transient increase in GI‐AEs is similar to what has been observed in other AOM trials [16, 19]. As mentioned earlier, only a minority of the participants reported a daily intake of fiber‐containing meal products at baseline. Acarbose delays the digestion of carbohydrates, which leads to an increase in enzymatic activity further down in the small intestine [24]. If the dose escalation rate is too rapid in regard to the ramping up enzymes, carbohydrates spill over to the colon, which triggers bloating and flatulence [24]. A slower and more flexible dose escalation schedule, together with proper dietary support, would likely have dampened the tolerability issues observed in the initial phase of the trial in participants with GI issues [24]. In contrast to other AOMs, there were minimal occurrences of nausea, vomiting, and constipation. Of note, the participants in the EMP16 group seem to have not been substantially affected by the side effects, as self‐reported quality of life was clearly improved. As with all other trials with EMP16, no SAEs occurred in the trial.

One limitation of the trial is the lack of diversity; almost all participants were White Caucasians, a common problem in many clinical trials [16, 17, 19]. However, there are no indications that orlistat efficacy should differ between different populations [10]. Acarbose may have a slightly higher weight loss effect in Eastern populations compared with Western populations [9], but the observed difference was very small in that study. Another limitation was the designation of categorical weight loss as a co‐primary endpoint. Sample size estimation was based on the relative weight change from the baseline endpoint alone and the trial was therefore not powered to fully address the categorical endpoint. As this was a phase 2 trial, prespecifying the relative weight change from baseline as the primary endpoint exclusively would have been more appropriate considering the performed sample size estimation.

## 5 Conclusion

This trial shows that acarbose has a meaningful contribution to the clinically relevant weight loss effect of EMP16, with additional improvements in important health markers. No serious safety issues were observed. EMP16 seems to be a promising new medication for long‐term obesity control and warrants further investigation in phase 3 trials.

## Author Contributions

All authors took part in designing the study outline. Helena Litorp was the signatory principal investigator of the clinical trial and responsible for site 1 (Uppsala, Sweden). Sandra Kuusk was the principal writer of the clinical trial protocol and the clinical trial report. Joakim Englund was the principal writer of the statistical analytical plan and responsible for statistical analyses. Stefan Grudén and Göran Alderborn were responsible for the pharmaceutical development of EMP16. Ulf Holmbäck wrote the first draft of the manuscript. All authors have taken part in writing, reviewing, and finalizing the manuscript. All authors have had access to the data and guarantee the validity of the presented data.

## Conflicts of Interest

Ulf Holmbäck, Stefan Grudén, Arvid Söderhäll, Göran Alderborn, and Anders Forslund have equity interests in Empros Pharma AB and have acted as employees or consultants for the company. Ulf Holmbäck, Stefan Grudén, Göran Alderborn and Anders Forslund hold the patent for the studied antiobesity medication. Sandra Kuusk, Helena Litorp, and Joakim Engblom are employed at or consultants for Clinical Trial Consultants AB but declare no conflicts of interest.

## Supporting information


**Data S1** Supporting Information.

## Data Availability

The data that support the findings of this study are available from the corresponding author upon reasonable request.
