# Supplementary material for: The Supportive Effect of Acarbose to Orlistat in Weight Management—A Randomized, Double‐Blind, Multiarm Phase 2 Trial
Source: Obesity (Silver Spring). 2025 Aug 6;33(10):1855–64. doi: 10.1002/oby.24369 (PMC12477108; doi:10.1002/oby.24369)
Supplement: Supplementary file 1 — Data S1 Supporting Information. [file OBY-33-1855-s001.docx]

## Supplemental Information

**The Supportive effect of Acarbose to Orlistat in Weight Management - a randomized, double-blind, multi-arm phase 2 trial**

Ulf Holmbäck^1*^, Stefan Grudén^2^, Sandra Kuusk^3^, Helena Litorp^3,4,5^, Joakim Englund^3^, Arvid Söderhäll^2^, Göran Alderborn^6^, Anders Forslund^4^

^1^ Department of Public Health and Caring Sciences, Uppsala University, Uppsala, Sweden

^2^ Empros Pharma AB, Solna, Sweden

^3^ Clinical Trial Consultants AB, Uppsala, Sweden

^4^ Department of Women’s and Children’s Health, Uppsala University, Uppsala, Sweden

^5^ Department of Global Public Health, Karolinska Institutet, Stockholm, Sweden

^6^ Department of Pharmaceutical Biosciences, Uppsala University, Uppsala, Sweden

Content

[Supplemental Information 1](#_Toc201826461)

[Description of EMP16 2](#_Toc201826462)

[Methodology 2](#_Toc201826463)

[Supplemental Table S1 Dose escalation schedule 3](#_Toc201826464)

[Outcomes 4](#_Toc201826465)

[Inclusion criteria 6](#_Toc201826466)

[Exclusion criteria 6](#_Toc201826467)

[Results 8](#_Toc201826468)

[Supplemental Table S2 Lifestyle (diet, sleep, and physical activity) questionnaire 8](#_Toc201826469)

[Supplemental Table S3 Meal pattern 9](#_Toc201826470)

[Supplemental Table S4 Baseline and change from baseline to end of study in TFEQ 10](#_Toc201826471)

[Supplemental Table S5 Baseline and change from baseline to end of study in PHQ-9 and GAD-7 10](#_Toc201826472)

[Supplemental Table S6 Adverse events by system organ class and preferred term 11](#_Toc201826473)

[Supplemental Table S7 Prevalence of adverse events of special interest during the trial 16](#_Toc201826474)

[Supplemental Table S8 Baseline and change from baseline to end of study in liver enzyme values 17](#_Toc201826475)

[References 17](#_Toc201826476)

## Description of EMP16

EMP16 is composed of three pharmaceutical fractions (granules denoted G1, G2 and G3) of multiple unit pellets in one capsule with different release rates and amounts of acarbose and orlistat.

1. G1 contains most of the acarbose dose that starts to release its drug load after approximately 30 minutes in the gastric phase to ensure proper mixing and continues to release API for 4-5 hours. The aim with the G1 component is to maintain the reduced gastric emptying with acarbose and, through proper mixing, minimize the side-effects seen with conventional acarbose and increase efficacy.
2. G2 contains most of the orlistat dose and a significant amount of the acarbose dose. G2 starts to dissolve in the distal duodenum. Thus, when G2 enters the duodenum, a burst of both APIs is released. The are several aims with this component: 1) reduce and delay the digestion and absorption of fat and carbohydrates; 2) by minimizing the release of orlistat prior to the duodenum, the unfavourable effects on gastric emptying time and appetite increase seen with conventional orlistat are avoided; 3) together with the G1 component to reduce insulin secretion, which in turn is hypothesized to have a favourable effect on fat storage.
3. G3 contains a small amount of orlistat, which starts to release small amounts of orlistat in the stomach and continues to release orlistat for several hours. The aim of this component is to support the G2 orlistat and help maintain reduced fat digestion throughout the GI tract.

## Methodology

This was a randomized, double-blind trial in participants with overweight or obesity. Prior to any trial assessments, participants were asked to provide signed informed consent to participate in the trial. There were 6 outpatient visits to the research clinics (Visits 1, 2, 3, 5, 7 and 9), and 3 telephone check-ups (Visits 4, 6 and 8). In addition, text reminders regarding investigated medical product (IMP) intake and dietary restrictions were sent between Visit 7 and Visit 8 and between Visit 8 and Visit 9. There were no overnight stays at the clinic.

Screening (Visit 1) took place within 35 days prior to the first dose. Eligible and consenting participants arrived at the research clinic in a fasting condition (at least 8 hours fasting) in the morning of the first dosing day (Visit 2). A re-check of eligibility including a symptom driven physical examination was performed as were measurements of weight, body composition, blood pressure and heart rate. Blood samples (fasting) were taken and quality of life, lifestyle, level of anxiety, depression, and personality traits were assessed. Breakfast was served at the clinic after blood sampling and assessments of body composition. The first dose of IMP was taken at home, in association with dinner.

Participants were randomized to either the three main arms:

- EMP16: 120 mg modified release orlistat/40 mg modified release acarbose, 80 participants.
- MR-O: 120 mg modified release orlistat, 80 participants.
- Conv-O: 120 mg orlistat in its conventional dosage form (Xenical®), 80 participants.

or the two smaller exploratory arms (to be presented in another publication)

- EMP16 60 mg orlistat/20 mg acarbose, 40 participants.
- Placebo, 40 participants

All participants followed a fixed dose escalation schedule (Supplemental Table S1).

### Supplemental Table S1 Dose escalation schedule

| **Arm** | **Week 1 to 2** | **Week 3 to 4** | **Week 5 to 26** |
| --- | --- | --- | --- |
| EMP16-120/40 | 60 mg orlistat/20 mg acarbose  (1 capsule per day) | 60 mg orlistat/20 mg acarbose  (1 capsule TID) | 60 mg O/20 mg A (2 capsules TID) |
| MR-O | 60 mg MR-O  (1 capsule per day) | 60 mg MR-O (1 capsule TID) | 60 mg MR-O (2 capsules TID) |
| Conv-O | 60 mg Conv-O  (1 capsule per day) | 60 mg Conv-O (1 capsule TID) | 120 mg Conv-O plus placebo  (1 capsule of each TID) |

Note: Alli® was used as conventional orlistat during week 1 to 4.

After the 4-week dose escalation period, a 22-week treatment and observation period commenced.

During week 1 and 2, the IMP was taken in association with dinner only. On all other treatment days, the IMP was taken half-way through each meal (breakfast, lunch, and dinner, respectively). The IMP was taken with approximately 50 mL to 200 mL water.

Participants visited the clinic at week 4, week 10, week 18, and week 26 corresponding to Visit 3, Visit 5, Visit 7, and Visit 9 for efficacy and safety/tolerability assessments. All visits to the clinic were performed in the morning and participants arrived in the fasted state (at least 8 hours fasting). Each visit comprised a physical examination (if indicated), blood sampling (fasting), and assessment of body weight/body composition. AE/concomitant medication collection was performed, and the Lifestyle questionnaire (Visits 3, 5 and 7), or all questionnaires (Visit 9), were completed. Breakfast was served at all visits to the clinic once blood sampling (fasting) and weight and body composition assessments had been performed. The participants took their IMP (brought from home) halfway through breakfast at Visits 3, 5, 7 and 9.

New IMP was handed out to the participants at Visits 2, 3, 5 and 7. Participants were asked to bring IMP bottles (including empty bottles) to Visits 3, 5, 7 and 9 for compliance calculations.

Telephone calls at week 7, 14, and 22 (corresponding to Visits 4, 6 and 8) for collection of AEs and use of concomitant medications were done between the outpatient visits, and text reminders were sent to motivate participants.

## Outcomes

Anthropometry: Weight, height, BMI, waist circumference and sagittal abdominal diameter

Body composition: Body fat percentage was measured using a bio-impedance measuring device (Tanita RD-545), according to the manufacturer’s instructions.

Blood pressure and heart rate

Blood samples: HbA1c (Hemoglobin A1c), glucose, insulin, total cholesterol, LDL (Low-density lipoprotein), HDL (High-density lipoprotein), TGs (triglycerides), ApoA1 (Apolipoprotein A1), ApoB (Apolipoprotein B), hs-CRP (high sensitivity C-reactive protein), Albumin and the liver enzymes aspartate aminotransferase (AST), alanine aminotransferase (ALT), alkaline phosphatase (ALP) and gamma-glutamyl transferase (GGT).

Metabolic indices:

*Visceral adiposity index* (VAI)(1) was defined as:

WC=waist circumference, BMI=body mass index, TG=triglyceride levels, HDL= High-density lipoprotein

*Fatty Liver Index* (FLI)(2) was calculated as:

e^y^ / (1 + e^y^) × 100

Where y = 0.953 × ln(TG, mg/dL) + 0.139 × BMI, kg/m^2^ + 0.718 × ln (GGT, U/L) + 0.053 × WC, cm – 15.745

*The HOMA index* is a measure on insulin sensitivity defined as:

fasting insulin (μU/mL) × fasting blood glucose (mmol/L)/22.5

Quality of life questionnaires:

The RAND-36 health questionnaire comprises 36 questions (3). The questionnaire taps 8 health concepts: physical functioning, bodily pain, role limitations due to physical health problems, role limitations due to personal or emotional problems, emotional well-being, social functioning, energy/fatigue, and general health perception. Each question has 2, 3, 5 or 6 levels. The questionnaire also includes a single item that provides an indication of perceived change in health. A low total score indicates poor health-related quality of life while a high score indicates good health-related quality of life.

EQ-5D is a standardized instrument for measuring health-related quality of life developed by the EuroQol group (<https://euroqol.org/eq-5d-instruments>). The 5 level EQ-5D (EQ-5D-5L) consists of the EQ-5D descriptive system and the EQ visual analogue scale (EQ VAS). The descriptive system comprises 5 dimensions: mobility, self-care, usual activities, pain/discomfort, and anxiety/depression. Each dimension has 5 levels: no problems, slight problems, moderate problems, severe problems, and extreme problems. The participant is asked to indicate his/her health state by ticking the box next to the most appropriate statement in each of the 5 dimensions. This decision results in a 1-digit number that expresses the level selected for that dimension. The digits for the 5 dimensions can be combined into a 5-digit number that describes the participant’s health state. The EQ VAS records the participant’s self-rated health on a vertical VAS, where the endpoints are labelled ‘The best health you can imagine’ and ‘The worst health you can imagine’. The VAS can be used as a quantitative measure of health outcome that reflect the participant’s own judgement.

Lifestyle (diet, physical activity and sleep) questionnaire

The diet part of the questionnaire consisted of 5 multiple choice questions about dietary and eating habits.

1. How often do you eat vegetables or root vegetables (fresh, frozen or cooked)?
2. How often do you eat cookies, chocolate, sweets, chips or soft drinks?
3. How often do you eat whole meal bread or other fiber-rich products?
4. How often do you eat three meals (breakfast, lunch and dinner)?
5. How often do you eat hamburgers, pizza and other fast food?

Each question was scored 0 to 3, i.e., the maximum score was 15. A low score indicated that the participant had unhealthy eating habits while a high score indicated that the participant followed the recommendations for healthy eating habits. The questionnaire was modified from the food index questions used by the Swedish National Board of Health and Welfare (4).

The physical activity part of the questionnaire consisted of 3 multiple choice questions

1. On average in the past month, how many minutes did you engage in heart-rate-elevating moderate physical activity?
2. On average in the past month, how many minutes did you engage in heart-rate-elevating intense physical activity?
3. On average in the past month, how often have you performed muscle-strengthening physical activity involving the body's major muscle groups?

Each question was scored 0 to 3, i.e., the maximum score was 12.

The sleep part of the questionnaire consisted of 2 multiple choice questions about sleeping habits.

1. How many hours have you slept on average in the last week?
2. To what extent does disturbed sleep constitute a health problem for you?

The questions were scored 0 to 3 and 0 to 4, respectively, i.e., the maximum score was 7. A low score indicated that the participant had sleeping problems while a high score indicated that the participant had normal sleeping habits.

Eating Questionnaire

The self-rating TFEQ was developed to measure cognitive and behavioral components of eating (5). The instrument contains 36 items with a yes/no response format, 14 items on a 1-4 response scale and 1 vertical rating.

Anxiety and depression questionnaires

GAD-7 is a self-reported questionnaire for screening and measuring the severity of generalized anxiety disorder (GAD, <https://adaa.org/sites/default/files/GAD-7_Anxiety-updated_0.pdf> . The assessment is indicated by a total score which is calculated by adding together the scores from 0 to 3 in 7 questionnaire items evaluated over the past 2 weeks.

The PHQ-9 questionnaire is a multi-purpose instrument for screening, diagnosing, monitoring and measuring the severity of depression (<https://www.apa.org/depression-guideline/patient-health-questionnaire.pdf>). The questionnaire incorporates Diagnostic and Statistical Manual of Mental Disorders (DSM)-IV diagnostic criteria for depression together with other leading major depressive symptoms intro a brief self-reporting tool. A total score was calculated according to instructions provided in the questionnaire.

## Inclusion criteria

For inclusion in the trial, the participants had to fulfil the following criteria:

1. Willing and able to give written informed consent for participation in the trial.
2. Males or females aged ≥18 years.
3. BMI ≥ 30 or ≥ 27 kg/m² in the presence of other risk factors based on participant interview e.g., hypertension (either or not treated with antihypertensive agents), glucose dysregulation (defined as elevated fasting glucose ≥6.1 mmol/L or HbA1c >42mmol/mol), T2DM that was treated with lifestyle changes (no medication allowed), and/or dyslipidemia (either or not treated with antihyperlipidemic agents). If indicated, plasma/serum total cholesterol, LDL, HDL, and/or TGs were measured to verify eligibility as judged by the Investigator.
4. No clinically significant abnormalities regarding physical examination, vital signs, electrocardiogram (ECG), and laboratory values at the time of the screening visit, as judged by the Investigator.
5. Adequate renal function: creatinine <1.5 times upper limit of normal (ULN).
6. Adequate hepatic function: aspartate aminotransferase (AST), alanine aminotransferase (ALT), alkaline phosphatase (ALP), and gamma-glutamyl transferase (GGT) <2.5 times ULN and bilirubin <1.5 times ULN.

## Exclusion criteria

Potential participants were not allowed to enter the trial if any of the following exclusion criteria were fulfilled:

1. Weight unstable (≥ 5% reported change during the previous 3 months) preceding screening and randomization.
2. Subjects who were pregnant, who were currently breastfeeding, who intended to become pregnant within the period of the trial, or who had given birth within the 6 months preceding the screening visit.
3. T2DM treated with medication.
4. History or presence of any clinically significant disease, disorder, or history of surgery which, in the opinion of the Investigator, could have either put the participant at risk because of participation in the trial, or influenced the results or the participant’s ability to participate in the trial including but not limited to:
5. GI problems/diseases, e.g., diseases that affect intestinal absorption and peristalsis such as inflammatory bowel diseases, irritable bowel syndrome (IBS), and Hirschsprung’s disease.
   1. Cholestasis.
   2. Chronical malabsorption syndrome.
   3. Severe allergic, cardiac, or hepatic disease.
   4. Previous GI surgery that might influence GI function significantly, such as previous bariatric surgery, and previous gallbladder surgery as judged by the investigator.
6. Potential participants with well-treated chronic diseases (e.g., celiac disease and lactose intolerance) could be included in the trial at the discretion of the Investigator.
7. Significant clinical illness within the preceding 2 weeks of the first administration of IMP at the discretion of the Investigator.
8. Any significant medical/surgical procedure or trauma within 4 weeks of the first administration of IMP at the discretion of the Investigator.
9. Any planned major surgery within the duration of the trial.
10. Any use of drugs altering glucose metabolism and drugs used for diabetes (A10A and A10B) or drugs that are affected by, or that affect, orlistat and acarbose, within 2 weeks prior to the first administration of IMP.
11. Regular use of prescribed or non-prescribed medication within 2 weeks prior to the first administration of IMP as judged by the Investigator. Potential participants who were on stable treatment with anti-depressants (e.g., selective serotonin re-uptake inhibitors [SSRI]) for at least 2 months could be included at the discretion of the Investigator.
12. Untreated high blood pressure (systolic blood pressure >160 mmHg and diastolic blood pressure >100 mmHg at the screening visit).
13. Known hypersensitivity to any of the test substances.
14. Malignancy within the past 5 years with the exception of in situ removal of basal cell carcinoma.
15. Excessive intake of alcohol, as judged by the Investigator.
16. Current or history of alcohol abuse and/or use of anabolic steroids or drugs of abuse.
17. Positive screen for drugs of abuse, or positive screen for alcohol, at the screening visit (Visit 1).
18. Any positive result at the screening visit (Visit 1) for serum hepatitis B surface antigen, hepatitis C antibody and Human Immunodeficiency Virus (HIV).
19. Plasma donation within 1 month of the screening visit (Visit 1) or any blood donation (or corresponding blood loss) during the 3 months prior to the screening visit.
20. Administration of another new chemical entity (defined as a compound which has not been approved for marketing) or has participated in any other clinical trial that included drug treatment within 3 months of the first administration of IMP in this trial. Participants consented and screened but not dosed in previous trials were not excluded.
21. Investigator considered the potential participant unlikely to comply with trial procedures, restrictions, and requirements.

## Results

### Supplemental Table S2 Lifestyle (diet, sleep, and physical activity) questionnaire

| Lifestyle questionnaire | EMP16 | MR-O | Conv-O |
| --- | --- | --- | --- |
| Diet summary score | 9.1 (2.5) | 8.7 (2.3) | 8.7 (2.4) |
| Change from baseline, mean (95% CI) | 2.4 (1.9 to 2.9) | 3.0 (2.3 to 3.6) | 2.3 (1.8 to 2.8) |
| Physical activity summary score | 3.8 (2.8) | 3.6 (2.5) | 4.0 (2.9) |
| Change from baseline, mean (95% CI) | 0.5 (-0.3 to 1.2) | 1.0 (0.4 to 1.6) | 0.2 (-0.5 to 1.0) |
| Sleep summary score | 4.4 (1.6) | 4.0 (1.6) | 4.4 (1.6) |
| Change from baseline, mean (95% CI) | -0.2 (-0.6 to 0.1) | 0.3 (0.0 to 0.6) | -0.1 (-0.5 to 0.3) |

### Supplemental Table S3 Meal pattern

|  | | EMP16 | MR-O | Conv-O |
| --- | --- | --- | --- | --- |
| How often do you eat vegetables or root vegetables (fresh, frozen or cooked)? | | | | |
| Baseline | Once a week or less | 5/80 (6.3%) | 9/80 (11%) | 7/80 (8.8%) |
|  | A few times a week | 26/80 (33%) | 28/80 (35%) | 21/80 (26%) |
|  | Once every day | 36/80 (45%) | 26/80 (33%) | 38/80 (48%) |
|  | Twice every day or more | 13/80 (16%) | 17/80 (21%) | 14/80 (18%) |
| End of study | Once a week or less | 2/66 (3.0%) | 0 | 4/73 (5.5%) |
|  | A few times a week | 15/66 (23%) | 14/69 (20%) | 12/73 (16%) |
|  | Once every day | 30/66 (45%) | 31/69 (45%) | 31/73 (42%) |
|  | Twice every day or more | 19/66 (29%) | 24/69 (35%) | 26/73 (36%) |
| How often do you eat cookies, chocolate, sweets, chips or soft drinks? | | | | |
| Baseline | Once a week or less | 24/80 (30%) | 23/80 (29%) | 8/80 (10%) |
|  | A few times a week | 37/80 (46%) | 42/80 (53%) | 47/80 (59%) |
|  | Once every day | 16/80 (20%) | 13/80 (16%) | 20/80 (25%) |
|  | Twice every day or more | 3/80 (3.8%) | 2/80 (2.5%) | 5/80 (6.3%) |
| End of study | Once a week or less | 37/66 (56%) | 40/69 (58%) | 26/73 (36%) |
|  | A few times a week | 26/66 (39%) | 27/69 (39%) | 42/73 (58%) |
|  | Once every day | 3/66 (4.5%) | 2/69 (2.9%) | 5/73 (6.8%) |
|  | Twice every day or more | 0 | 0 | 0 |
| How often do you eat whole meal bread or other fiber-rich products? | | | | |
| Baseline | Once a week or less | 19/80 (24%) | 22/80 (28%) | 13/80 (16%) |
|  | A few times a week | 34/80 (43%) | 35/80 (44%) | 41/80 (51%) |
|  | Once every day | 21/80 (26%) | 19/80 (24%) | 18/80 (23%) |
|  | Twice every day or more | 6/80 (7.5%) | 4/80 (5.0%) | 8/80 (10%) |
| End of study | Once a week or less | 7/66 (11%) | 4/69 (5.8%) | 6/73 (8.2%) |
|  | A few times a week | 21/66 (32%) | 23/69 (33%) | 24/73 (33%) |
|  | Once every day | 22/66 (33%) | 28/69 (41%) | 32/73 (44%) |
|  | Twice every day or more | 16/66 (24%) | 14/69 (20%) | 11/73 (15%) |
| How often do you eat three meals (breakfast, lunch and dinner)? | | | | |
| Baseline | Once a week or less | 8/80 (10%) | 13/80 (16%) | 6/80 (7.5%) |
|  | A few times a week | 11/80 (14%) | 13/80 (16%) | 14/80 (18%) |
|  | Once every day | 30/80 (38%) | 29/80 (36%) | 33/80 (41%) |
|  | Twice every day or more | 31/80 (39%) | 25/80 (31%) | 27/80 (34%) |
| End of study | Once a week or less | 0 | 1/69 (1.4%) | 1/73 (1.4%) |
|  | A few times a week | 3/66 (4.5%) | 5/69 (7.2%) | 5/73 (6.8%) |
|  | Once every day | 17/66 (26%) | 20/69 (29%) | 22/73 (30%) |
|  | Twice every day or more | 46/66 (70%) | 43/69 (62%) | 45/73 (62%) |
| How often do you eat hamburgers, pizza and other fast food? | | | | |
| Baseline | Once a week or less | 34/80 (43%) | 28/80 (35%) | 27/80 (34%) |
|  | A few times a week | 23/80 (29%) | 18/80 (23%) | 21/80 (26%) |
|  | Once every day | 23/80 (29%) | 34/80 (43%) | 28/80 (35%) |
|  | Twice every day or more | 0 | 0 | 4/80 (5.0%) |
| End of study | Once a week or less | 46/66 (70%) | 53/69 (77%) | 48/73 (66%) |
|  | A few times a week | 2/66 (3.0%) | 3/69 (4.3%) | 5/73 (6.8%) |
|  | Once every day | 18/66 (27%) | 13/69 (19%) | 20/73 (27%) |
|  | Twice every day or more | 0 | 0 | 0 |

### Supplemental Table S4 Baseline and change from baseline to end of study in TFEQ

| TFEQ-21 | EMP16 | MR-O | Conv-O |
| --- | --- | --- | --- |
| Uncontrolled eating domain score, mean (SD) | 2.2 (0.7) | 2.3 (0.7) | 2.3 (0.6) |
| Change from baseline, mean (95% CI) | -0.2 (-0.3 to 0.0) | -0.2 (-0.4 to -0.1) | -0.2 (-0.4 to -0.1) |
| Cognitive restraint domain score, mean (SD) | 2.1 (0.6) | 2.0 (0.6) | 2.0 (0.5) |
| Change from baseline, mean (95% CI) | 0.5 (0.3 to 0.6) | 0.5 (0.4 to 0.7) | 0.4 (0.3 to 0.6) |
| Emotional eating domain score, mean (SD) | 2.2 (0.9) | 2.2 (0.9) | 2.2 (0.8) |
| Change from baseline, mean (95% CI) | -0.2 (-0.3 to 0.0) | -0.3 (-0.4 to -0.1) | -0.2 (-0.4 to -0.1) |

### Supplemental Table S5 Baseline and change from baseline to end of study in PHQ-9 and GAD-7

|  | EMP16 | MR-O | Conv-O |
| --- | --- | --- | --- |
| PHQ-9, Depression Severity, mean (SD) | 5.2 (3.8) | 5.5 (4.5) | 5.5 (4.3) |
| Change from baseline, mean (95% CI) | -1.2 (-2.1 to -0.3) | -1.7 (-2.7 to -0.7) | -1.3 (-2.1 to -0.5) |
| GAD-7, Total score, mean (SD) | 3.3 (3.3) | 3.4 (3.5) | 3.0 (3.4) |
| Change from baseline, mean (95% CI) | -0.9 (-1.5 to -0.3) | -0.9 (-1.7 to 0.0) | -0.9 (-1.7 to 0.0) |

### Supplemental Table S6 Adverse events by system organ class and preferred term

|  | EMP16 (N=80) | | MR-O (N=80) | | Conv-O (N=80) | |
| --- | --- | --- | --- | --- | --- | --- |
| System organ class  Preferred term | n (%) | m | n (%) | m | n (%) | m |
| **Total** | **78 (98%)** | **334** | **73 (91%)** | **277** | **75 (94%)** | **281** |
| **Gastrointestinal disorders** | **72 (90%)** | **233** | **66 (83%)** | **169** | **68 (85%)** | **169** |
| Abdominal distension | 13 (16%) | 15 | 9 (11%) | 9 | 5 (6.3%) | 7 |
| Abdominal pain | 5 (6.3%) | 7 | 6 (7.5%) | 6 | 5 (6.3%) | 5 |
| Abdominal pain upper | 4 (5.0%) | 7 | 5 (6.3%) | 5 | 6 (7.5%) | 6 |
| Abnormal feces | 0 | 0 | 0 | 0 | 1 (1.3%) | 1 |
| Anal incontinence* | 19 (24%) | 22 | 10 (13%) | 12 | 11 (14%) | 11 |
| Breath odor | 0 | 0 | 1 (1.3%) | 1 | 0 | 0 |
| Constipation | 1 (1.3%) | 1 | 1 (1.3%) | 1 | 2 (2.5%) | 2 |
| Defecation urgency | 3 (3.8%) | 3 | 4 (5.0%) | 5 | 8 (10%) | 11 |
| Diarrhea | 50 (63%) | 57 | 51 (64%) | 67 | 53 (66%) | 58 |
| Dry mouth | 0 | 0 | 0 | 0 | 2 (2.5%) | 2 |
| Dyspepsia | 2 (2.5%) | 3 | 2 (2.5%) | 2 | 1 (1.3%) | 1 |
| Eructation | 1 (1.3%) | 1 | 0 | 0 | 0 | 0 |
| Feces discolored | 0 | 0 | 0 | 0 | 0 | 0 |
| Feces hard | 2 (2.5%) | 2 | 1 (1.3%) | 1 | 0 | 0 |
| Flatulence | 46 (58%) | 49 | 24 (30%) | 24 | 15 (19%) | 15 |
| Food poisoning | 0 | 0 | 1 (1.3%) | 1 | 1 (1.3%) | 1 |
| Frequent bowel movements | 1 (1.3%) | 1 | 1 (1.3%) | 1 | 1 (1.3%) | 1 |
| Gastroesophageal reflux disease | 2 (2.5%) | 2 | 1 (1.3%) | 1 | 0 | 0 |
| Hematochezia | 2 (2.5%) | 2 | 0 | 0 | 0 | 0 |
| Hemorrhoids | 0 | 0 | 0 | 0 | 1 (1.3%) | 1 |
| Nausea | 4 (5.0%) | 4 | 1 (1.3%) | 1 | 5 (6.3%) | 5 |
| Proctalgia | 0 | 0 | 2 (2.5%) | 2 | 1 (1.3%) | 1 |
| Rectal discharge* | 33 (41%) | 38 | 22 (28%) | 26 | 25 (31%) | 29 |
| Steatorrhea | 15 (19%) | 15 | 4 (5.0%) | 4 | 10 (13%) | 10 |
| Toothache | 3 (3.8%) | 3 | 0 | 0 | 1 (1.3%) | 1 |
| Vomiting | 1 (1.3%) | 1 | 0 | 0 | 1 (1.3%) | 1 |
| **Infections and infestations** | **33 (41%)** | **38** | **39 (49%)** | **53** | **35 (44%)** | **47** |
| Borrelia infection | 1 (1.3%) | 2 | 0 | 0 | 0 | 0 |
| Bronchitis | 0 | 0 | 1 (1.3%) | 1 | 0 | 0 |
| COVID-19 | 0 | 0 | 4 (5.0%) | 4 | 1 (1.3%) | 1 |
| Conjunctivitis | 0 | 0 | 1 (1.3%) | 1 | 1 (1.3%) | 1 |
| Diverticulitis | 0 | 0 | 0 | 0 | 1 (1.3%) | 1 |
| Ear infection | 0 | 0 | 2 (2.5%) | 2 | 0 | 0 |
| Gastroenteritis | 0 | 0 | 1 (1.3%) | 1 | 0 | 0 |
| Gastroenteritis viral | 2 (2.5%) | 2 | 0 | 0 | 3 (3.8%) | 3 |
| Hand-foot-and-mouth disease | 1 (1.3%) | 1 | 0 | 0 | 1 (1.3%) | 1 |
| Herpes zoster | 1 (1.3%) | 1 | 0 | 0 | 0 | 0 |
| Influenza | 3 (3.8%) | 3 | 0 | 0 | 1 (1.3%) | 1 |
| Localized infection | 0 | 0 | 0 | 0 | 0 | 0 |
| Lyme disease | 1 (1.3%) | 1 | 0 | 0 | 2 (2.5%) | 2 |
| Nasopharyngitis | 23 (29%) | 25 | 32 (40%) | 40 | 26 (33%) | 31 |
| Oral herpes | 0 | 0 | 0 | 0 | 0 | 0 |
| Otitis media | 1 (1.3%) | 1 | 0 | 0 | 0 | 0 |
| Pneumonia | 1 (1.3%) | 1 | 1 (1.3%) | 1 | 0 | 0 |
| Respiratory tract infection | 0 | 0 | 0 | 0 | 1 (1.3%) | 1 |
| Respiratory tract infection viral | 0 | 0 | 0 | 0 | 0 | 0 |
| Rhinitis | 0 | 0 | 0 | 0 | 0 | 0 |
| Sinusitis | 0 | 0 | 1 (1.3%) | 1 | 1 (1.3%) | 1 |
| Skin infection | 1 (1.3%) | 1 | 0 | 0 | 0 | 0 |
| Tonsillitis | 0 | 0 | 0 | 0 | 2 (2.5%) | 2 |
| Tooth infection | 0 | 0 | 1 (1.3%) | 1 | 0 | 0 |
| Upper respiratory tract infection | 0 | 0 | 1 (1.3%) | 1 | 1 (1.3%) | 1 |
| Urinary tract infection | 0 | 0 | 0 | 0 | 1 (1.3%) | 1 |
| **Nervous system disorders** | **14 (18%)** | **19** | **10 (13%)** | **12** | **9 (11%)** | **13** |
| Dizziness | 3 (3.8%) | 3 | 0 | 0 | 1 (1.3%) | 1 |
| Headache | 13 (16%) | 13 | 7 (8.8%) | 8 | 8 (10%) | 11 |
| Migraine | 1 (1.3%) | 1 | 3 (3.8%) | 4 | 1 (1.3%) | 1 |
| Nerve compression | 1 (1.3%) | 1 | 0 | 0 | 0 | 0 |
| Sciatica | 0 | 0 | 0 | 0 | 0 | 0 |
| Somnolence | 0 | 0 | 0 | 0 | 0 | 0 |
| Syncope | 1 (1.3%) | 1 | 0 | 0 | 0 | 0 |
| **Musculoskeletal and connective tissue disorders** | **6 (7.5%)** | **7** | **8 (10%)** | **8** | **10 (13%)** | **10** |
| Arthralgia | 2 (2.5%) | 2 | 1 (1.3%) | 1 | 2 (2.5%) | 2 |
| Back pain | 2 (2.5%) | 2 | 4 (5.0%) | 4 | 1 (1.3%) | 1 |
| Bursitis | 0 | 0 | 0 | 0 | 0 | 0 |
| Coccydynia | 0 | 0 | 0 | 0 | 1 (1.3%) | 1 |
| Muscle spasms | 0 | 0 | 0 | 0 | 2 (2.5%) | 2 |
| Myalgia | 1 (1.3%) | 2 | 0 | 0 | 1 (1.3%) | 1 |
| Osteoarthritis | 0 | 0 | 1 (1.3%) | 1 | 0 | 0 |
| Pain in extremity | 0 | 0 | 0 | 0 | 1 (1.3%) | 1 |
| Plantar fasciitis | 0 | 0 | 2 (2.5%) | 2 | 1 (1.3%) | 1 |
| Rotator cuff syndrome | 0 | 0 | 0 | 0 | 1 (1.3%) | 1 |
| Tendonitis | 1 (1.3%) | 1 | 0 | 0 | 0 | 0 |
| Torticollis | 0 | 0 | 0 | 0 | 0 | 0 |
| **General disorders and administration site conditions** | **2 (2.5%)** | **2** | **7 (8.8%)** | **9** | **4 (5.0%)** | **4** |
| Chest discomfort | 0 | 0 | 0 | 0 | 0 | 0 |
| Cyst | 0 | 0 | 1 (1.3%) | 2 | 0 | 0 |
| Exercise tolerance decreased | 0 | 0 | 1 (1.3%) | 1 | 0 | 0 |
| Fatigue | 1 (1.3%) | 1 | 0 | 0 | 3 (3.8%) | 3 |
| Feeling cold | 0 | 0 | 0 | 0 | 0 | 0 |
| Hunger | 0 | 0 | 1 (1.3%) | 1 | 0 | 0 |
| Inflammation | 0 | 0 | 1 (1.3%) | 1 | 0 | 0 |
| Malaise | 0 | 0 | 2 (2.5%) | 2 | 0 | 0 |
| Pyrexia | 1 (1.3%) | 1 | 2 (2.5%) | 2 | 0 | 0 |
| Sensation of foreign body | 0 | 0 | 0 | 0 | 0 | 0 |
| Thirst | 0 | 0 | 0 | 0 | 1 (1.3%) | 1 |
| **Metabolism and nutrition disorders** | **2 (2.5%)** | **2** | **7 (8.8%)** | **9** | **6 (7.5%)** | **6** |
| Decreased appetite | 1 (1.3%) | 1 | 4 (5.0%) | 4 | 1 (1.3%) | 1 |
| Food craving | 0 | 0 | 1 (1.3%) | 1 | 0 | 0 |
| Gout | 0 | 0 | 1 (1.3%) | 2 | 0 | 0 |
| Hyperinsulinemia | 0 | 0 | 1 (1.3%) | 1 | 0 | 0 |
| Hyperlipidemia | 0 | 0 | 0 | 0 | 1 (1.3%) | 1 |
| Hypertriglyceridemia | 1 (1.3%) | 1 | 0 | 0 | 2 (2.5%) | 2 |
| Increased appetite | 0 | 0 | 1 (1.3%) | 1 | 0 | 0 |
| Iron deficiency | 0 | 0 | 0 | 0 | 1 (1.3%) | 1 |
| Type 2 diabetes mellitus | 0 | 0 | 0 | 0 | 1 (1.3%) | 1 |
| **Injury, poisoning and procedural complications** | **5 (6.3%)** | **5** | **1 (1.3%)** | **1** | **1 (1.3%)** | **1** |
| Animal bite | 0 | 0 | 0 | 0 | 0 | 0 |
| Ankle fracture | 0 | 0 | 0 | 0 | 0 | 0 |
| Arthropod sting | 1 (1.3%) | 1 | 0 | 0 | 0 | 0 |
| Bite | 1 (1.3%) | 1 | 0 | 0 | 0 | 0 |
| Carbon monoxide poisoning | 1 (1.3%) | 1 | 0 | 0 | 0 | 0 |
| Fall | 0 | 0 | 0 | 0 | 0 | 0 |
| Fibula fracture | 1 (1.3%) | 1 | 0 | 0 | 0 | 0 |
| Joint dislocation | 0 | 0 | 0 | 0 | 0 | 0 |
| Ligament sprain | 0 | 0 | 0 | 0 | 1 (1.3%) | 1 |
| Limb injury | 1 (1.3%) | 1 | 0 | 0 | 0 | 0 |
| Muscle strain | 0 | 0 | 1 (1.3%) | 1 | 0 | 0 |
| Post-traumatic pain | 0 | 0 | 0 | 0 | 0 | 0 |
| Road traffic accident | 0 | 0 | 0 | 0 | 0 | 0 |
| **Skin and subcutaneous tissue disorders** | **4 (5.0%)** | **6** | **2 (2.5%)** | **2** | **3 (3.8%)** | **3** |
| Alopecia | 1 (1.3%) | 2 | 0 | 0 | 1 (1.3%) | 1 |
| Erythema | 0 | 0 | 0 | 0 | 1 (1.3%) | 1 |
| Hyperhidrosis | 1 (1.3%) | 1 | 0 | 0 | 0 | 0 |
| Pruritus | 1 (1.3%) | 2 | 0 | 0 | 1 (1.3%) | 1 |
| Rash | 1 (1.3%) | 1 | 1 (1.3%) | 1 | 0 | 0 |
| Urticaria | 0 | 0 | 1 (1.3%) | 1 | 0 | 0 |
| **Psychiatric disorders** | **1 (1.3%)** | **1** | **2 (2.5%)** | **2** | **4 (5.0%)** | **4** |
| Acute stress disorder | 0 | 0 | 0 | 0 | 0 | 0 |
| Anxiety | 0 | 0 | 0 | 0 | 0 | 0 |
| Depressed mood | 1 (1.3%) | 1 | 0 | 0 | 0 | 0 |
| Depression | 0 | 0 | 0 | 0 | 1 (1.3%) | 1 |
| Insomnia | 0 | 0 | 0 | 0 | 1 (1.3%) | 1 |
| Sleep disorder | 0 | 0 | 0 | 0 | 1 (1.3%) | 1 |
| Stress | 0 | 0 | 2 (2.5%) | 2 | 1 (1.3%) | 1 |
| **Vascular disorders** | **3 (3.8%)** | **4** | **0** | **0** | **3 (3.8%)** | **3** |
| Hypertension | 2 (2.5%) | 3 | 0 | 0 | 3 (3.8%) | 3 |
| Hypotension | 1 (1.3%) | 1 | 0 | 0 | 0 | 0 |
| **Surgical and medical procedures** | **4 (5.0%)** | **4** | **3 (3.8%)** | **3** | **3 (3.8%)** | **5** |
| Dental operation | 0 | 0 | 0 | 0 | 1 (1.3%) | 1 |
| Eye operation | 0 | 0 | 0 | 0 | 1 (1.3%) | 2 |
| Foot operation | 1 (1.3%) | 1 | 0 | 0 | 0 | 0 |
| Hip surgery | 0 | 0 | 0 | 0 | 1 (1.3%) | 1 |
| Knee operation | 1 (1.3%) | 1 | 0 | 0 | 0 | 0 |
| Limb operation | 0 | 0 | 1 (1.3%) | 1 | 0 | 0 |
| Nail operation | 1 (1.3%) | 1 | 0 | 0 | 0 | 0 |
| Shoulder operation | 0 | 0 | 0 | 0 | 1 (1.3%) | 1 |
| Tooth extraction | 1 (1.3%) | 1 | 1 (1.3%) | 1 | 0 | 0 |
| Wisdom teeth removal | 0 | 0 | 1 (1.3%) | 1 | 0 | 0 |
| **Ear and labyrinth disorders** | **3 (3.8%)** | **3** | **0** | **0** | **4 (5.0%)** | **5** |
| Aural polyp | 0 | 0 | 0 | 0 | 1 (1.3%) | 1 |
| Ear pain | 0 | 0 | 0 | 0 | 0 | 0 |
| Motion sickness | 0 | 0 | 0 | 0 | 1 (1.3%) | 1 |
| Vertigo | 3 (3.8%) | 3 | 0 | 0 | 3 (3.8%) | 3 |
| **Investigations** | **2 (2.5%)** | **2** | **2 (2.5%)** | **2** | **4 (5.0%)** | **4** |
| Alanine aminotransferase increased | 0 | 0 | 1 (1.3%) | 1 | 0 | 0 |
| Aspartate aminotransferase increased | 0 | 0 | 0 | 0 | 1 (1.3%) | 1 |
| Blood glucose decreased | 1 (1.3%) | 1 | 0 | 0 | 0 | 0 |
| Blood glucose increased | 1 (1.3%) | 1 | 0 | 0 | 0 | 0 |
| Blood pressure increased | 0 | 0 | 0 | 0 | 1 (1.3%) | 1 |
| Heart rate decreased | 0 | 0 | 1 (1.3%) | 1 | 0 | 0 |
| Hepatic enzyme increased | 0 | 0 | 0 | 0 | 2 (2.5%) | 2 |
| **Reproductive system and breast disorders** | **2 (2.5%)** | **2** | **4 (5.0%)** | **5** | **1 (1.3%)** | **1** |
| Dysmenorrhea | 1 (1.3%) | 1 | 3 (3.8%) | 3 | 1 (1.3%) | 1 |
| Heavy menstrual bleeding | 0 | 0 | 1 (1.3%) | 1 | 0 | 0 |
| Intermenstrual bleeding | 1 (1.3%) | 1 | 0 | 0 | 0 | 0 |
| Menstruation delayed | 0 | 0 | 0 | 0 | 0 | 0 |
| Scrotal mass | 0 | 0 | 1 (1.3%) | 1 | 0 | 0 |
| **Renal and urinary disorders** | **0** | **0** | **0** | **0** | **2 (2.5%)** | **2** |
| Calculus urinary | 0 | 0 | 0 | 0 | 0 | 0 |
| Hematuria | 0 | 0 | 0 | 0 | 1 (1.3%) | 1 |
| Micturition urgency | 0 | 0 | 0 | 0 | 0 | 0 |
| Pollakiuria | 0 | 0 | 0 | 0 | 1 (1.3%) | 1 |
| Renal colic | 0 | 0 | 0 | 0 | 0 | 0 |
| **Respiratory, thoracic and mediastinal disorders** | **3 (3.8%)** | **3** | **1 (1.3%)** | **1** | **1 (1.3%)** | **1** |
| Cough | 0 | 0 | 1 (1.3%) | 1 | 0 | 0 |
| Dyspnea | 1 (1.3%) | 1 | 0 | 0 | 0 | 0 |
| Nasal congestion | 0 | 0 | 0 | 0 | 0 | 0 |
| Oropharyngeal pain | 2 (2.5%) | 2 | 0 | 0 | 1 (1.3%) | 1 |
| **Immune system disorders** | **3 (3.8%)** | **3** | **0** | **0** | **1 (1.3%)** | **1** |
| Hypersensitivity | 1 (1.3%) | 1 | 0 | 0 | 0 | 0 |
| Immunization reaction | 2 (2.5%) | 2 | 0 | 0 | 0 | 0 |
| Seasonal allergy | 0 | 0 | 0 | 0 | 1 (1.3%) | 1 |
| **Hepatobiliary disorders** | **0** | **0** | **0** | **0** | **1 (1.3%)** | **1** |
| Biliary colic | 0 | 0 | 0 | 0 | 1 (1.3%) | 1 |
| **Cardiac disorders** | **0** | **0** | **1 (1.3%)** | **1** | **0** | **0** |
| Palpitations | 0 | 0 | 1 (1.3%) | 1 | 0 | 0 |
| **Endocrine disorders** | **0** | **0** | **0** | **0** | **0** | **0** |
| Hyperglycemia | 0 | 0 | 0 | 0 | 0 | 0 |
| **Eye disorders** | **0** | **0** | **0** | **0** | **1 (1.3%)** | **1** |
| Dry eye | 0 | 0 | 0 | 0 | 1 (1.3%) | 1 |

* “Anal incontinence” is the preferred term for fecal incontinence and “Rectal discharge” is the preferred term for oily spotting.

### Supplemental Table S7 Prevalence of adverse events of special interest during the trial

|  | | EMP16 | MR-O | Conv-O | *P*- value^1^ (EMP16 versus MR-O) | *P*- value (EMP16 versus Conv-O) |
| --- | --- | --- | --- | --- | --- | --- |
| **Fecal incontinence** | | | | | | |
| Visit 3 Week 4 | No | 70/77 (91%) | 73/78 (94%) | 74/78 (95%) | 0.534 | 0.343 |
|  | Yes | 7/77 (9.1%) | 5/78 (6.4%) | 4/78 (5.1%) |  |  |
| Visit 4 Week 7 | No | 61/74 (82%) | 70/75 (93%) | 70/75 (93%) | 0.074 | 0.057 |
|  | Yes | 13/74 (18%) | 5/75 (6.7%) | 5/75 (6.7%) |  |  |
| Visit 5 Week 10 | No | 60/74 (81%) | 68/72 (94%) | 69/74 (93%) | 0.035 | 0.023 |
|  | Yes | 14/74 (19%) | 4/72 (5.6%) | 5/74 (6.8%) |  |  |
| Visit 6 Week 14 | No | 64/72 (89%) | 68/72 (94%) | 71/74 (96%) | 0.285 | 0.083 |
|  | Yes | 8/72 (11%) | 4/72 (5.6%) | 3/74 (4.1%) |  |  |
| Visit 7 Week 18 | No | 64/69 (93%) | 69/70 (99%) | 70/73 (96%) | 0.191 | 0.271 |
|  | Yes | 5/69 (7.2%) | 1/70 (1.4%) | 3/73 (4.1%) |  |  |
| Visit 8 Week 22 | No | 65/68 (96%) | 67/69 (97%) | 72/73 (99%) | 0.719 | 0.267 |
|  | Yes | 3/68 (4.4%) | 2/69 (2.9%) | 1/73 (1.4%) |  |  |
| Visit 9 Week 26 | No | 61/66 (92%) | 68/69 (99%) | 71/73 (97%) | 0.174 | 0.732 |
|  | Yes | 5/66 (7.6%) | 1/69 (1.4%) | 2/73 (2.7%) |  |  |
| Overall | No | 59/77 (77%) | 70/78 (90%) | 68/78 (87%) | 0.108 | 0.066 |
|  | Yes | 18/77 (23%) | 8/78 (10%) | 10/78 (13%) |  |  |
| **Oily spotting** | | | | | | |
| Visit 3 Week 4 | No | 61/77 (79%) | 71/78 (91%) | 63/78 (81%) | 0.044 | 0.810 |
|  | Yes | 16/77 (21%) | 7/78 (9.0%) | 15/78 (19%) |  |  |
| Visit 4 Week 7 | No | 50/74 (68%) | 58/75 (77%) | 56/75 (75%) | 0.208 | 0.438 |
|  | Yes | 24/74 (32%) | 17/75 (23%) | 19/75 (25%) |  |  |
| Visit 5 Week 10 | No | 49/74 (66%) | 57/72 (79%) | 55/74 (74%) | 0.042 | 0.259 |
|  | Yes | 25/74 (34%) | 15/72 (21%) | 19/74 (26%) |  |  |
| Visit 6 Week 14 | No | 51/72 (71%) | 57/72 (79%) | 56/74 (76%) | 0.118 | 0.454 |
|  | Yes | 21/72 (29%) | 15/72 (21%) | 18/74 (24%) |  |  |
| Visit 7 Week 18 | No | 49/69 (71%) | 56/70 (80%) | 56/73 (77%) | 0.101 | 0.319 |
|  | Yes | 20/69 (29%) | 14/70 (20%) | 17/73 (23%) |  |  |
| Visit 8 Week 22 | No | 55/68 (81%) | 57/69 (83%) | 59/73 (81%) | 0.543 | 0.712 |
|  | Yes | 13/68 (19%) | 12/69 (17%) | 14/73 (19%) |  |  |
| Visit 9 Week 26 | No | 54/66 (82%) | 57/69 (83%) | 60/73 (82%) | 0.515 | 0.550 |
|  | Yes | 12/66 (18%) | 12/69 (17%) | 13/73 (18%) |  |  |
| Overall | No | 44/77 (57%) | 57/78 (73%) | 53/78 (68%) | 0.092 | 0.428 |
|  | Yes | 33/77 (43%) | 21/78 (27%) | 25/78 (32%) |  |  |

^1^ Mixed effects logistic regression with repeated measures analysis, using multiple imputation of missing data from visits 4 and onwards.

### Supplemental Table S8 Baseline and change from baseline to end of study in liver enzyme values

|  | EMP16 | MR-O | Conv-O |
| --- | --- | --- | --- |
| Alanine Aminotransferase (ukat/L), mean (SD) | 0.59 (0.35) | 0.61 (0.53) | 0.58 (0.31) |
| Change from baseline, mean (95% CI) | -0.02 (-0.09 to 0.05) | -0.09 (-0.20 to 0.01) | -0.01 (-0.07 to 0.05) |
| Alkaline Phosphatase (ukat/L), mean (SD) | 1.17 (0.35) | 1.08 (0.27) | 1.21 (0.35) |
| Change from baseline, mean (95% CI) | 0.19 (0.13 to 0.24) | 0.21 (0.17 to, 0.24) | 0.27 (0.20 to 0.33) |
| Aspartate Aminotransferase (ukat/L), mean (SD) | 0.46 (0.16) | 0.47 (0.17) | 0.46 (0.13) |
| Change from baseline, mean (95% CI) | -0.02 (-0.05 to 0.01) | -0.02 (-0.06 to 0.01) | 0.00 (-0.03, 0.03) |
| Gamma Glutamyl Transferase (ukat/L), mean (SD) | 0.68 (0.57) | 0.57 (0.39) | 0.61 (0.38) |
| Change from baseline, mean (95% CI) | -0.12 (-0.22 to -0.01) | -0.07 (-0.13 to -0.01) | 0.04 (-0.05 to 0.13) |

## References

1. Amato MC, Giordano C, Galia M, Criscimanna A, Vitabile S, Midiri M*, et al.* Visceral Adiposity Index: a reliable indicator of visceral fat function associated with cardiometabolic risk. *Diabetes Care* 2010;**33:** 920-922.

2. Bedogni G, Bellentani S, Miglioli L, Masutti F, Passalacqua M, Castiglione A*, et al.* The Fatty Liver Index: a simple and accurate predictor of hepatic steatosis in the general population. *BMC Gastroenterol* 2006;**6:** 33.

3. Hays RD, Morales LS. The RAND-36 measure of health-related quality of life. *Annals of Medicine* 2001;**33:** 350-357.

4. Sepp H, Ekelund U, Becker W. *Enkätfrågor om kost och fysisk aktivitet bland vuxna: underlag till urval av frågor i befolkningsinriktade enkäter [Questionnaires on diet and physical activity among adults: basis for selection of questions in population-oriented surveys]*. Livsmedelsverket: Uppsala, 2004.

5. Karlsson J, Persson L-O, Sjöström L, Sullivan M. Psychometric properties and factor structure of the Three-Factor Eating Questionnaire (TFEQ) in obese men and women. Results from the Swedish Obese Subjects (SOS) study. *International Journal of Obesity* 2000;**24:** 1715-1725.
